# A Feasibility Study Using a Machine Learning Suicide Risk Prediction Model Based on Open-Ended Interview Language in Adolescent Therapy Sessions

**DOI:** 10.3390/ijerph17218187

**Published:** 2020-11-05

**Authors:** Joshua Cohen, Jennifer Wright-Berryman, Lesley Rohlfs, Donald Wright, Marci Campbell, Debbie Gingrich, Daniel Santel, John Pestian

**Affiliations:** 1 Clarigent Health, 5412 Courseview Drive, Suite 210, Mason, OH 45040, USA; lrohlfs@clarigenthealth.com (L.R.); dwright@clarigenttech.com (D.W.); mcampbell@clarigenthealth.com (M.C.); 2 Department of Social Work, College of Allied Health Sciences, University of Cincinnati, Cincinnati, OH 45221, USA; wrigh2jb@ucmail.uc.edu; 3 The Children’s Home, 5050 Madison Road, Cincinnati, OH 45227, USA; dgingrich@bestpoint.org; 4 Department of Pediatrics, Division of Biomedical Informatics, Cincinnati Children’s Hospital Medical Center, Cincinnati, OH 45229, USA; Daniel.Santel@cchmc.org (D.S.); John.Pestian@cchmc.org (J.P.)

**Keywords:** machine learning, natural language processing, suicidal risk, risk assessment, mental health, therapy, suicidal ideation

## Abstract

Background: As adolescent suicide rates continue to rise, innovation in risk identification is warranted. Machine learning can identify suicidal individuals based on their language samples. This feasibility pilot was conducted to explore this technology’s use in adolescent therapy sessions and assess machine learning model performance. Method: Natural language processing machine learning models to identify level of suicide risk using a smartphone app were tested in outpatient therapy sessions. Data collection included language samples, depression and suicidality standardized scale scores, and therapist impression of the client’s mental state. Previously developed models were used to predict suicidal risk. Results: 267 interviews were collected from 60 students in eight schools by ten therapists, with 29 students indicating suicide or self-harm risk. During external validation, models were trained on suicidal speech samples collected from two separate studies. We found that support vector machines (AUC: 0.75; 95% CI: 0.69–0.81) and logistic regression (AUC: 0.76; 95% CI: 0.70–0.82) lead to good discriminative ability, with an extreme gradient boosting model performing the best (AUC: 0.78; 95% CI: 0.72–0.84). Conclusion: Voice collection technology and associated procedures can be integrated into mental health therapists’ workflow. Collected language samples could be classified with good discrimination using machine learning methods.

## 1. Introduction

Suicide rates among adolescents have risen steadily over the last decade, and suicide is now the second leading cause of death among 10–34 year olds [1]. In settings where suicidal thoughts and behaviors are assessed, such as mental health centers, traditional methods for evaluating risk employ survey screening tools, such as the Patient Health Questionnaire 9 [2] and the Columbia Suicide Severity Rating Scale [3]. Although these scales are frequently used and have been widely tested [2,3,4,5,6,7,8,9,10], assessed accuracy of suicide risk is often subject to both the rater’s intuition and the responder’s ability to answer the questions while in distress. Youth in particular may have difficulty responding to such screeners, for reasons such as social desirability [11], lack of engagement with the rater [12], and lack of understanding [13]. Therefore, exploring more objective approaches to identifying youth at risk for suicide is warranted. Additionally, the dynamic and fluid state of suicidality [14] can be challenging to measure with static screeners. A person’s mental state’s nuances are too idiosyncratic for measurement tools often tested with homogenous populations. Instead, suicide risk data collection should be derived from the content of thoughts of the individual’s experience.

Speech is one of the most complex human activities [15], coordinating diverse brain regions, and is affected by physical, neurological, and mental health conditions [16]. Prior research has shown how machine learning models can classify these conditions based on the linguistic and acoustic markers in speech [16,17,18,19,20,21,22]. Underlying machine learning models’ success is that these conditions cause neurophysiological changes that can be consistently measured with voice data (linguistic and acoustic markers) [17,21,23]. While much of the brain’s structure–function relationship remains unknown [24], studies on the brains of those with suicide attempts or who died by suicide have found notable differences compared to controls, including a decrease in gray matter and activity changes of specific brain regions [25,26].

Machine learning (ML) has emerged as a method by which data from human characteristics, such as speech [16,17], physical and social media activity [27], and electronic medical records [28,29], can be analyzed in higher concentration and with better precision. Natural language processing (NLP) has been previously used to identify mental health and suicide-related states using both written and spoken samples, and it has shown that, in addition to content words (what we say), function words (how we say it) are also important to language identification [18,19,30,31,32,33]. Often during these classification tasks, language from controls (those without a condition) and cases (those with a condition) is turned into a vector representing the frequency words—or sequences of words—occurring in each language sample. These vectors are then used to “train” ML models to recognize patterns and create rules that allow for discrimination between cases and controls. The different types of ML models (e.g., support vector machines and extreme gradient boosting) approach the same goal of classifying language as case or control as accurately as possible using different mathematical methods, leading to the emergence of unique rules to accomplish this task.

After an ML model is trained, different evaluation strategies and metrics are used to evaluate performance on data that was not used to train the model [34,35]. During validation, new language vectors are shown to the ML model. Given unknown data, the trained ML model returns the probability for a sample belonging to a target class (i.e., case). This result can then be compared to the actual class (i.e., what is known about that language sample) to determine the performance of the ML model. A preferred performance metric for evaluating ML models is the area under the receiver operating characteristic curve (AUC) [34], which may be interpreted as the probability that a randomly selected case will receive a greater probability for belonging to the case group than a randomly selected control [36]. An AUC of 0.5 represents a model that predicts as well as random chance, and an AUC of 1.0 is a perfect model. Many mental health diagnostic checklists and inventories perform with AUCs under clinically realistic conditions in the range of 0.7–0.8 [36,37].

Previous research explored using NLP to classify suicide risk. In 2016, Pestian et al. performed the Adolescent Controlled Trial (ACT) with 60 adolescents admitted to a large, urban, pediatric emergency department (ED) with suicidal complaints (case) or orthopedic injuries (control) [18]. They completed the Columbia Suicide Severity Rating Scale (C-SSRS) and a semi-structured interview based on characteristics of suicidality (called the Ubiquitous Questionnaire, UQ). The UQ was designed to elicit language for machine learning model training [18]. Resulting transcripts were analyzed with a combined NLP/ML approach, which successfully classified 58 of the 60 participants (96.7%) [18].

Expanding on the ACT, the Suicide Thought Markers (STM) Study recruited 379 adults and children across three sites [19]. The procedure was similar to the previous study; however, participants with mental illness were also included with the suicidal and control cohorts [19]. Results from this study suggested that the NLP/ML method identified suicidal people from the interview transcripts with over 90% accuracy [19]. Specifically, classifiers trained on interview transcripts performed with an AUC of 0.87 ± 0.02 when classifying suicidal thoughts and behaviors versus those with and without mental illness, and an AUC of 0.93 ± 0.02 when classifying suicidal thoughts and behaviors versus controls without mental illness, using a leave-one-interview-out cross-validation technique [19].

All suicidal participants in the ACT and STM studies demonstrated a risk for suicide that led to their admission to the ED or a psychiatric unit [18,19]. Participants’ suicide-related thoughts and behaviors ranged from suicide-related ideations to suicide-related behaviors, including self-harm (type I and II) and suicide attempt (type I and II) [38], with over 75% of suicidal STM participants scoring ≥ 4 on the C-SSRS’s intensity of suicidal ideation scale [3,20]. Therefore, models trained on this language aim to identify those within this range of risk for suicide.

Due to limited innovation and person-centered measurement tools in suicide risk assessment, machine learning, specifically NLP, is timely. This method of both data collection and analysis offers an objective and less biased approach to identifying people with suicidal thoughts and behaviors (STBs). While this study procedure has been successfully implemented to identify these individuals in a variety of settings, such as the ED, in- and outpatient clinics [18,19], and in a recent study of individuals with epilepsy and psychiatric comorbidities [21], it has yet to be implemented as part of outpatient mental health therapy sessions. This feasibility study was conducted in partnership with a child and adolescent mental health agency to understand how this technology integrates into a mental health professional’s (MHP) workflow with adolescents and if the collected language samples can be analyzed with ML methods to predict risk for suicide. Overall, we found MHPs were accepting of the technology and procedures, and ML models trained on language samples from the ACT and STM studies performed well when predicting suicide risk in this new population.

## 2. Methods

This study’s objectives were to (1) explore the feasibility of incorporating previous study procedures to capture the language and predict the level of suicidal risk into mental health therapy sessions, and (2) evaluate if machine learning methods accurately identify level of suicide risk by classifying language.

All subjects gave their informed consent and assent for inclusion before they participated in the study. The study was conducted in accordance with the Declaration of Helsinki, and the protocol was approved by the Ethics Committee of Cincinnati Children’s Hospital Medical Center Institutional Review Board 2019-0391 (project identification code).

### 2.1. Participants and Setting

#### 2.1.1. Mental Health Professionals

Ten licensed mental health professionals (MHPs) from eight schools in three school districts in a Midwestern urban city in the United States participated. MHPs were recruited through a collaboration with local mental health agencies that primarily serve children and adolescents. The MHPs recruited client participants from among their existing caseloads (adolescents already in therapy) being seen at school during school hours for various mental and behavioral health conditions.

**Figure 1:**
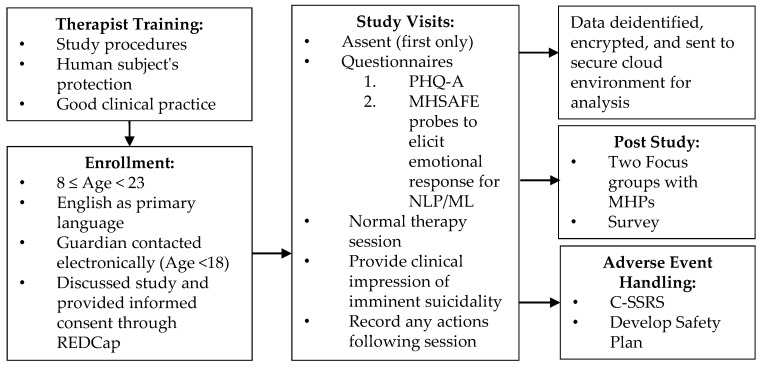
Schematic of study procedure.

MHPs attended one of two training sessions where the smartphone app, called MHSAFE (renamed from the Ubiquitous Questions in previous studies: Hope, Secrets, Anger, Fears, and Emotional Pain), was installed on their smartphones (iOS and Android). During the training sessions, the MHPs learned the study procedures and participated in the human subject’s protection and good clinical practice training, outlined in Figure 1.

#### 2.1.2. Adolescent Clients

The criteria for adolescent recruitment were: (1) currently receiving services from a mental health agency at a school, outpatient, or at a college/university health services center, (2) age ≥ 8 years and < 23 years, (3) able to provide informed consent or parental permission and assent, (4) age 8–18 must have parental permission to participate in the study, and (5) English as a primary language. At the first therapy session for the study (most participants had a prior relationship with their therapist), the MHPs introduced the study to their clients. Parents were contacted electronically (text or email) to arrange a telephone call to discuss the study and review the consent process. Informed consent was completed via REDCap software [39,40]. Participants provided assent through the MHSAFE app during their first study visit.

The participants and MHPs received a $25.00 gift card for their time in the study.

### 2.2. Study Procedure

During therapy sessions, the participant’s MHP administered the Patient Health Questionnaire 9-Item Modified for Adolescents (PHQ-A) and the MHSAFE probes. Figure 1 outlines these study procedures. The PHQ-9 is a rigorously tested, reliable, and valid instrument for depression in adolescents, with a sensitivity and specificity of 89.5% and 77.5%, respectively, corresponding with a threshold score ≥ 11 out of 27 [41]. The PHQ-A has two more suicide-related questions than the PHQ-9 and has not been widely tested for suicidal risk in youth, though scores on the PHQ-9, especially item 9, are a strong predictor of suicide attempts and death by suicide [4,5,6,7,8]. However, in comparative trials, the Columbia Suicide Severity Rating Scale (C-SSRS) has shown to be a stronger predictor as a full scale of suicide risk than the single question on the PHQ-9 [9,10]. The MHSAFE probes are modeled after the UQ, described in previous work [18,19,42,43]. In brief, the MHSAFE probes are a semi-structured, 5–10 min open interview process designed to elicit an emotional response from participants by asking about their hopes, secrets, anger, fear, and emotional pain. Following the therapy session, MHPs entered their clinical impression of the client’s mental state into the app, rating the participant on a 0–100 scale on imminent suicide risk compared to a population baseline. This clinical impression was developed from the MHP’s best clinical judgment during the session.

The MHSAFE app was used to record the entire therapy session. The audio files were manually transcribed and diarized (speaker identified) using a HIPAA compliant service that reports 99% accuracy. The conversation segments containing the probes were manually identified by two reviewers trained to identify the beginning and ending of the probe segments from the full therapy session transcript.

### 2.3. Data Analysis

All analysis was performed using the Python programming language (version 3.7.5) [44]. The open source Python libraries Pandas (version 1.1.2) [45,46], Numpy (version 1.18.5) [47,48], scikit-learn (version 0.23.2) [49], Matplotlib (version 3.7.5) [50], SciPy (version 1.5.2) [51], NLTK (version 3.2.2) [52], spaCy (version 3.0.0a16) [53], and XGBoost (v. 0.90) [54] were used for data analysis and all NLP/ML model building.

The number of probes asked during the interviews was determined automatically using word vectors to find semantically similar words to the five areas of the probes [53,55]. The counts were then validated by a single reviewer trained to manually assess the number of probes present in a transcript.

The NLP/ML pipeline used in this study followed similar techniques used by Pestian et al., focused on the term frequency of n-grams (contiguous sequence of n number of words) [18,19,20,21]. The text was normalized by expanding contractions and lemmatizing (replacing words by their root) [52]. N-grams were then vectorized to be fed into ML models. Due to the many words spoken and the size of n-grams analyzed, the language vectors were large (>1000 dimensions). Because not every n-gram will meaningfully influence a model’s output, the language vectors’ size can be reduced. Scikit-learn’s SelectKBest function was used to identify features with the highest ANOVA F-value [49], with the number of features selected as a tunable hyperparameter to optimize model performance.

Previous work focused primarily on support vector machines (SVMs) [18,19,20,21]; however, we also explored the performance of logistic regression (LR) and extreme gradient boosting (XGB) models. SVM models have demonstrated excellent performance in previous tasks classifying suicidal language from semi-structured interviews, perform well in high-dimensional spaces, and resist overfitting [18,19,20,21]. During SVM tuning, hyperparameters considered include: the regularization parameter (C), the kernel (radial basis function and linear kernels), the kernel coefficient (gamma, if applicable), and the class weight [49]. LR is a popular machine learning model for classification because it is relatively simple. During LR tuning, hyperparameters considered include: the inverse of regularization strength (C), the algorithm used during optimization, and the class weight [49]. For extreme gradient tree boosting, the XGBoost system was used, which has given state-of-the-art results on various problems [54]. During XGB tuning, hyperparameters considered include: the minimum child weight, the minimum loss reduction required to partition a leaf (gamma), step size shrinkage (eta), the subsample of the training instances, and the maximum depth of a tree [56]. These different ML models may allow for the development of unique rules and potential interactions among classification features.

During model training, the only input was the participant’s language, labeled as case or control. During model testing, a participant’s language was fed into the model, and a probability for belonging to the case group was returned. Model performance was then evaluated by comparing model predictions to the participant’s labeled group (case or control). The model’s AUC was used as the primary evaluation criteria for model performance. AUC confidence intervals (CI) were calculated using the DeLong method [57].

### 2.4. Machine Learning Model Performance on Training Data (Internal Validation)

Data from the ACT and STM studies have been internally validated in separate publications and report AUCs from 0.69–0.93, depending on the features (acoustic or linguistic) or participants included (control, those with mental illness not suicidal, those without mental illness and suicidal) [18,19]. The best performing model used an SVM with only linguistic features to classify between suicidal and non-mentally ill controls for adults and adolescents [19]. The lowest performing model used acoustics (e.g., fundamental frequency and pause lengths) to differentiate between suicidal adolescents from mentally ill adolescents [19], which can be partially explained by the low variability of acoustic features for mental states along with known overlaps between acoustic markers for suicide, depression, and other mental illnesses [17]. Only linguistic features were considered in this study.

As mentioned, the ACT and STM studies were collected with similar procedures; however, this pilot differed to better accommodate fitting into the workflow of an MHP. As a baseline for model performance, a leave-one-site-out cross-validation technique was used with the ACT and STM study data, which included data from four sites: ACT, University of Cincinnati Medical Center (UCMC), Ohio; STM, UCMC; STM, Cincinnati Children’s Hospital Medical Center (CCMHC), Ohio; and STM, Princeton Community Hospital (PCH), West Virginia. We differentiated between the two UCMC sites because they were collected as part of separate studies with different participants at different times [18,19]. In this method, data from all sites except one were used to train the model, with data from the final site used as the test site to evaluate model performance. During training, model hyperparameters were tuned [58], including the number of features. This was done iteratively so that every site served as the test site, and highlighted model generalizability challenges and performance expectations across different sites. Table 1 displays a summary of the training data.

### 2.5. Machine Learning Model Performance on Pilot Data (External Validation)

For external validation, different machine learning algorithms were trained and tuned on subsections of the ACT and STM dataset (control, suicidal, or mentally ill), and then used to predict suicidal risk from the language samples collected in this pilot. The number of features (i.e., the number of n-grams) available to the classifier was used as a tunable hyperparameter, ranging from 5–2000 features. The suicidal risk for model performance evaluation was determined by answers to the PHQ-A, which has three items related to the immediacy of suicide risk and self-harm, shown in Table 2. Answers to item 9 and item 12 on the PHQ-A were used to identify cases (suicidal risk) and controls (no suicidal risk) in this study. This suicidal risk can be characterized by recent suicide- and death-related ideations.

## 3. Results

### 3.1. Population and Data Collection

Between April to August 2019, 10 therapists agreed to participate in the study and enrolled 60 participants. Participants attended 1–16 sessions, which resulted in a total of 267 recorded therapy sessions. The PHQ-A was collected in 249 (93%) sessions, and MHPs provided their ratings for imminent suicidal risk for every session. Participant demographics and general questionnaire results are found in Table 3.

Of those who completed the PHQ-A, 96 sessions reported some degree of suicide risk by answering positively to one or more of the three questions related to suicide on the PHQ-A, representing 29 participants. Total scores of 11 on the PHQ-A are often used as thresholds for clinically relevant depression measures in adolescents [41]. Over 31% of the PHQ-A measures suggested the presence of depression in the participants. During the study, six CSSRS-SF screeners were administered to further assess for suicide risk. Additional information can be found in Table S1.

Participants’ electronic medical records were also collected. Many participants had multiple mental health diagnoses: 46 had anxiety disorders, 4 had adolescent onset, 33 had mood disorders, 4 had development disorders, 3 had substance abuse, and 1 had a physical behavior diagnosis. Of the 60 participants, 43 were on medications.

### 3.2. Usage of the MHSAFE Probes

Table 4 shows a summary of the number of probes asked in each session, the average word count of those sessions, and the number of cases present. At least one of the five MHSAFE probes were asked in 264 (99%) therapy sessions, and at least three probes were asked in 247 (93%) of the sessions. On average, the MHSAFE probe segments were 11.8 ± 6.6 min, and transcripts of only the participant were 868 ± 795 words per session. However, 84% of the segments were less than 13 min, and 85% of the sessions were less than 1000 words. The high standard deviation of length and the word count may be attributed to therapists’ training to prioritize therapy over the study procedures. The MHSAFE probes reportedly revealed topics discussed for the majority of the session in ~15% of the sessions. The manually transcribed probe segments of the participant were used as linguistic features for the ML models.

### 3.3. Leave-One-Site-Out Validation with Training Data

**Figure 2:**
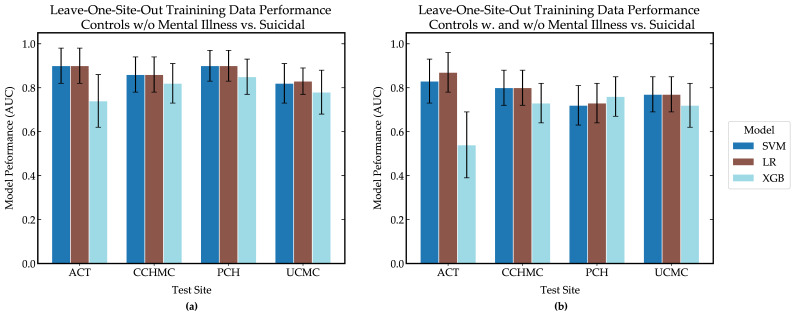
Leave-one-site-out results for training data with different machine learning (ML) models using (**a**) controls without mental illness and suicidal thoughts, and (**b**) controls with and without mental illness and suicidal thoughts. Error bars indicate a 95% confidence interval. ML models used include logistic regression (LR), support vector machines (SVM), and extreme gradient boosting (XGB). Studies and test sites include the ACT study (collected at UCMC) and the STM study collected at CCHMC, PCH, and UCMC.

Figure 2 shows leave-one-site-out cross-validation results for our training data for different ML models. In general, NLP/ML models were better able to discriminate between controls without mental illness and suicidal individuals (Figure 2a, AUC: 0.8–0.9) than a combination of controls with and without mental illness versus suicidal individuals (Figure 2b, AUC: 0.7–0.8), regardless of the ML model used and the site serving as the test site. This was consistent with previous studies using these data [18,19].

The logistic regression (LR) model performed the best on the training data when controls with mental illness were included (average AUC = 0.80; 95% CI = 0.82–0.88) and when they were not (average AUC = 0.87; 95% CI = 0.79–0.95). Support vector machines (SVMs) displayed similar discriminative ability as LR when controls with mental illness were included (average AUC = 0.78; 95% CI = 0.69–0.87) and when they were not (average AUC = 0.87; 95% CI = 0.79–0.95). Extreme gradient boosting (XGB) had lower discriminative ability when mentally ill controls were included (average AUC = 0.69; 95% CI = 0.58–0.80) and when they were not (average AUC = 0.80; 95% CI = 0.70–0.90). These performance ranges served as a baseline to compare against for language collected in this study.

### 3.4. Model Performance on Language Collected from Pilot

The best performing model was the extreme gradient boosting model (AUC = 0.78; 95% CI = 0.72–0.84) trained on controls without mental illness and suicidal language samples for predicting suicidal risk as identified by item 9 and item 12 on the PHQ-A, based on language collected in this study. The logistic regression (AUC = 0.76; 95% CI = 0.70–0.82) and support vector machine models (AUC = 0.75; 95% CI = 0.69–0.81) trained on controls without mental illness and suicidal language samples performed with slightly lower discriminative ability. Models that included the language samples from controls with mental illness in the training data had lower discriminative power than models trained without these language samples, as summarized in Table 5, along with the top five features for each model. The top features were determined from the training data, and their root replaced words (e.g., “am” is the first-person singular version of the verb “be”). Logistic regression and the support vector machine’s feature weights were positive or negative, indicating whether these features influenced the model’s prediction towards the case (+) or control (−). Extreme gradient boosting models’ feature importance is always positive and reflects how frequently a feature was used to make decisions.

### 3.5. Data Collected from Mental Health Professionals

Table 6 shows a summary for each MHP’s enrolled participants and suicide risk score statistics. We found that the top three MHPs enrolled 63% of the participants and produced 70% of the sessions, attributed primarily to varying caseloads and consent ratios. Table 6 also shows each MHP’s average suicidal risk score, along with their standard deviation and ranges. We found that the average score for all MHPs was 11.0 ± 8.3, with the maximum score of 70 corresponding to one of the most serious cases, in which mobile crisis support was contacted, and the participant was referred to the hospital. Several MHPs reported technical issues inputting their clinical impression scores into the app with a slider bar.

The MHPs also recorded relevant actions following therapy sessions. The majority (>94%) of sessions resulted in participants marked as “stable, resumed normal schedule.” One participant was sent home, parents were contacted seven times, MHPs consulted with their supervisor three times, safety plans were developed three times, two participants were referred to the hospital, and the mobile crisis was called once. The ~6% of cases where a participant was not marked as “stable, resumed normal schedule” may have been a result of these other actions or the MHP not recording actions following a session.

## 4. Discussion

In this study, we find integrating technology via a smartphone app into mental health therapy sessions and collecting language samples for machine learning models feasible. Models trained on language samples from separate studies that were not collected as part of a mental health therapy session were used to assess how well suicidal risk identified through the PHQ-A could be predicted based on language samples from this pilot. These techniques to capture the language and measure level of suicide risk using NLP and ML methods produced acceptable results, despite being collected in the less controlled environment of adolescent mental health therapy sessions compared to previous trials [18,19].

Clinical applications could grant MHPs a different perspective on a client’s level of suicide risk determined by their language, a more dynamic and person-centered characteristic than specific risk factors that do not meaningfully predict outcomes [59]. It would be reasonable for MHPs to ask the MHSAFE probes as part of regular therapy sessions or at specific intervals to assess congruence of their client’s language, standardized scales, and the MHP’s clinical impression. These data, when combined, may provide a more complete picture of a client’s mental state, and ultimately improve outcomes. In future clinical trials, MHPs will be provided a “dashboard” that displays all collected information entered about a client, with the aim of using the data to inform clinical decision-making. We intend to study how these data may be used clinically to assess and monitor the degree of suicide risk and related mental states over time, and how clinical decision-making is aligned with the dynamic changes of the client’s mental states.

While most of the MHSAFE probe segments were less than 13 minutes, it should be noted that the average interview time in the multi-site STM study was shorter (8.1 ± 4.5 min) [19]. During training in current trials, we now provide more specific guidelines on asking the probes to make them more concise, although, as noted, some MHPs reported voluntarily using the entire therapy session for the probes if they revealed details that warranted further discussion. We are also investigating model performance on clinical language samples without the MHSAFE probes to determine if the probes are needed for accurate classification; however, previous studies have found the probe responses statistically significant in a hierarchical classifier’s ability to discriminate suicidal and non-suicidal language elicited from the probes versus a combination of 11 other open-ended questions [43].

Of the ML models tested, the XGB model provided the best discriminative ability when evaluated on the language collected in this study. Interestingly, this model had the poorest discriminative power on all but one site during internal validation of the training data, as seen in Figure 2. XGB models can create more complex rules for classification than LR and SVM models, which can lead to the model learning from unimportant characteristics (i.e., overfitting). We see in Table 5 that LR and SVM models had the same top five features for each training group, while the XGB models’ top five features were the most unique. It should be noted the amount of training data varied in the creation of Figure 2, and it may be that when all of the training data was made available when evaluating model performance on language from this study, the XGB model was better able to identify important features and became more robust.

**Figure 3:**
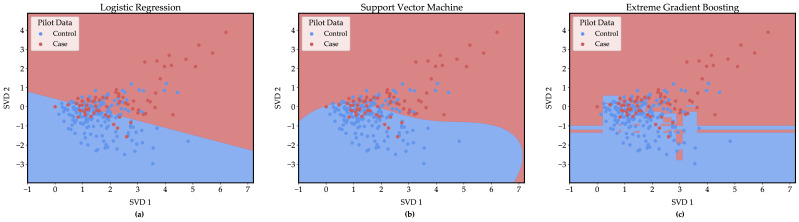
Decision boundaries for (**a**) logistic regression (LR), (**b**) support vector machine (SVM), and (**c**) extreme gradient boosting (XGB) models. Controls without mental illness and suicidal language samples from ACT and STM studies were dimensionally reduced using singular value decomposition. ML models were trained on dimensionally reduced language samples and used to classify coordinate points to create decision boundaries. The red and blue regions indicate coordinates that correspond to case and control classification, respectively. The red and blue points show dimensionally reduced language samples collected in this pilot. The LR model (**a**) shows the simplest rules used for classification and the XGB model (**c**) creates the most complex rules. Model performance indicated in these figures does not represent performance on non-dimensionally reduced data.

Figure 3 demonstrates the varying potential for complexity among LR, SVM (radial basis function kernel), and XGB models. Through a singular value decomposition (SVD), large language vectors that represent entire conversations can be reduced into two dimensions [51,60]. While some information is lost in this process and model performance is not fully represented in Figure 3, it may provide insights into model behavior. The red and blue regions of Figure 3 represent the coordinates learned from the training data (controls without mental illness and suicidal language) for classification as case or control, respectively, and where these regions meet is referred to as the decision boundary. The red and blue points represent language samples collected in this study. In Figure 3, the decision boundary for the LR (Figure 3a) and SVM (Figure 3b) models are smooth, continuous curves, while XGB’s (Figure 3c) decision boundary has more characteristics, emphasizing its capacity to create more complex, flexible rules for classification. As noted, NLP/ML techniques assume voice data is consistently changed by mental illness in measurable ways [17,23]. While in this study we have found a change of setting does not significantly impact model performance, it is likely that as these methods are extended to larger and more diverse groups of individuals, models like XGB that accommodate more complex rules will be required for accurate identification of suicidal risk based on language.

While machine learning models are often referred to as “black boxes” due to their overall technical complexity and lack of transparency into why specific predictions are made, new tools in explainable artificial intelligence (XAI) are being developed to answer this challenge [61,62,63]. Indeed, model interpretability will be essential for therapists and other users to trust and accept this technology, as well as to meet other ethical and regulatory considerations [61]. Future studies will employ these tools to focus on how specific features and feature interactions influence individual model predictions.

### Limitations and Lessons Learned

Some limitations should be noted. First, suicidal risk in this study is determined by the PHQ-A, a less accurate tool than the C-SSRS, and the reason for visit used in previous studies [18,19]. The PHQ-A does not discriminate between self-harm and passive thoughts of dying, and each question uses a different time frame reference (two weeks, last month, or whole life). Therefore, the suicidal risk may be overestimated in this sample, although it is also possible that some participants did not disclose suicidal thoughts or behaviors. An overestimation of suicide risk could result in clinical decisions that may not be aligned with the actual present risk. To correct for this, we have now included the C-SSRS short form screener version in each session to provide a more consistent, timely, and accurate standardized risk assessment. This will allow for better data validation during model development.

Second, because the goal of this pilot study was to understand how this tool can work in therapy sessions, some of the procedures were modified from the original ACT and STM studies, and the procedures were carried out at the discretion of the MHP. As noted, the MHSAFE probes are modeled after the UQ, but were altered to support generalizability across multiple settings. MHPs were not always consistent in how they administered the probes. Some began recording at the beginning of the session and stopped after the probes were completed. Some recorded the entire therapy session, and some waited to administer the probes at the end of the session and only began recording when asking the probes. MHPs also reported occasionally asking the probes with slight variations that may have been more age appropriate. For example, instead of asking about emotional pain, one MHP asked if there is “anything that’s really hurting your heart right now?” Going forward, after the pilot, we have revised the training, specifying to record the entire session and to administer the probes preferably at the beginning of therapy. However, we also continue to support flexibility with the therapist and the client’s needs for the session. A final limitation related to procedures was the use of the therapist impression slider rating system. The slider (1–100) was intended for the clinician to provide their impression of the client’s mental state, however, feedback from the clinicians about the slider was that it was not intuitive. The slider was investigator developed and not previously validated, therefore it was not used to assess model performance. We have modified this for future trials to reflect a five-point Likert scale with specific anchor descriptions to better rate the severity of the conditions.

Third, the technology, both with the smartphone app and voice collection, presented some difficulties. Therapists deployed the app on their personal or work phones, and occasionally there were issues with connectivity, app updates, or interruptions from other notifications. Manual transcriptionists reported challenges with a few of the audio files due to poor audio quality that may have been from background noise in the therapist’s office or if the phone was not placed in the optimal position for voice capture. While this likely did not significantly affect the resulting manual transcripts, for this technology to be scalable, this step will need to be automated using automatic speech recognition technology, with a performance that is dependent on audio quality [64,65]. We have worked to resolve these issues by improving the app technology, providing a version that can go on a therapist’s computer instead of their smartphone, and is better at instructing the therapists during training where to place the phone or microphone for optimal voice capture.

Lastly, this study was conducted with a single, regional, mental health partner, and the sample recruitment was limited to therapist participation and invitation of clients from their caseloads. A few therapists recorded a majority of the sessions. Although we were able to identify some successes and drawbacks of the process for this pilot, we anticipate that more concerns and barriers might arise when implementing on a broader scale. We are including feedback loops within the larger study design to make continual improvements to assist in maintaining the flow of the session while preserving the integrity of the data/data capture. We are also now recruiting nationally and working to increase diversity and inclusivity in our therapist sample.

## 5. Future Directions

As suggested in the limitations and lessons learned, we have made numerous modifications with the expectation of improving the research design and implementation for future studies. In these larger studies, we aim to collect data to continue to build models, as well as identify differences in language and acoustics related to suicide risk by person-level characteristics, such as gender, age, race, sexuality, and geographic location (dialect), and, additionally, the setting of the interview. We are also testing the use of the dashboard (described earlier) in clinical decision-making. Soon we anticipate providing a return-of-results from the ML models, with the idea being that clinicians will have real-time data output to make in-session decisions with the client. The vision is for the dashboard to be employed as a shared decision-making tool, where the client and the clinician may view the dashboard together to inform a collaborative and evolving treatment plan.

## 6. Conclusions

This study found that the implementation of a smartphone app to record speech in adolescent mental health therapy sessions is feasible. Previously developed procedures to elicit language samples for suicidal risk prediction machine learning models were adapted for use in therapy sessions. Machine learning models were trained on language collected from separate studies and used to predict suicide risk levels based on language collected in this study. These findings are an opportunity to implement new methods to support decision-making during a time of increased suicide and other mental health concerns. Lessons learned from the pilot have provided us a path forward to make improvements for a larger study.

## Figures and Tables

**Table 1:** Summary of machine learning model training data.

Site	No. Suicidal (%)	No. Mentally Ill (%)	No. Control (%)	Total (%)
ACT Study				
UCMC	30 (18.6)	0	30 (19.6)	60 (13.9)
STM Study				
UCMC	44 (27.5)	42 (33.3)	42 (27.5)	128 (29.2)
CCHMC	43 (26.9)	42 (33.3)	41 (26.8)	126 (28.7)
PCH	43 (26.9)	42 (33.3)	40 (26.1)	125 (28.5)
Total	160 (36.4)	126 (28.6)	153 (34.8)	439

**Table 2:** Suicide-related items on the Patient Health Questionnaire 9-Item Modified for Adolescents (PHQ-A).

PHQ-A Item	Question	Response Options
Item 9	How often in the past two weeks have you been bothered by thoughts that you would be better off dead, or thoughts of hurting yourself in some way?	Not at all (0), Several days (1), More than half the days (2), and Nearly every day (3)
Item 12	Has there been a time in the past month when you have had serious thoughts about ending your life?	Yes or No
Item 13	Have you EVER, in your WHOLE LIFE, tried to kill yourself or made a suicide attempt?	Yes or No

**Table 3:** Adolescent participant demographics and PHQ-A answer summaries.

			Sessions with Clinically Relevant Symptoms N = 249					Participants N = 60
	Participants	Sessions	PHQ-A ≥ 11	Item 9	Item 12	Item 13	Item 9 | Item 12 | Item 13	Item 9 | Item 12 | Item 13
**Count (%)**	60	267	77 (31)	68 (27)	39 (16)	59 (24)	96 (39)	29 (48)
**Average Age (years) (SD)**	12.8 (2.4)	12.5 (2.5)	13.6 (2.4)	13.7 (2.5)	14.7 (2.2)	13.8 (2.5)	13.5 (2.5)	13.5 (2.5)
**Male (%)**	50.0	41.6	28.6	33.8	35.9	59.3	39.6	37.9
**Race**								
**White (%)**	78.3	78.7	80.5	88.2	79.5	76.3	82.3	79.3
**Biracial or Multiracial (%)**	10.0	13.9	14.3	10.3	15.4	10.2	8.3	6.9
**Black/African American (%)**	8.3	5.6	3.9	0.0	0.0	8.5	5.2	6.9
**Not Reported (%)**	3.3	1.9	1.3	1.5	5.1	5.1	4.2	6.9

Note: Total scores ≥ 11 on the PHQ-A have been used for diagnosing depression with the greatest sensitivity and specificity in adolescents [41]. The suicide-related questions on the PHQ-A are broken out on a session and participant basis. The vertical bar | indicates a logical OR statement.

**Table 4:** Summary of MHSAFE probe usage.

No. of Probes Discussed	Zero	One	Two	Three	Four	Five
**No. of Sessions (%) N = 267**	3 (1.1)	5 (1.9)	11 (4.1)	20 (7.5)	29 (10.9)	198 (74.2)
**Full Session Average Participant Word Count (SD)**	532 (338)	1737 (1430)	1866 (1418)	1469 (947)	2117 (1430)	1721 (1182)
**Probe Segment Average Participant Word Count (SD)**	N/A	774 (611)	1438 (1020)	941 (690)	1051 (1079)	813 (740)
**No. of Sessions with PHQ-A (%) N = 249**	3 (1.2)	3 (1.2)	6 (2.4)	15 (6.0)	25 (10.0)	196 (78.7)
**No. of Cases (Item 9 | Item 12) (%) N = 70**	3 (4.3)	0 (0)	2 (2.9)	2 (2.9)	10 (14.3)	53 (75.7)

**Table 5:** Model performance predicting suicidal risk in pilot language data.

Model	AUC (95% CI)	Optimal No. of Features	Top 5 Features (Feature Importance or Weight)
**Training Data: Controls Without Mental Illness and Suicidal**			
**Extreme Gradient Boosting**	0.78 (0.72–0.84)	11	feel like, me angry, i be angry, no no, depression
**Logistic Regression**	0.76 (0.70–0.82)	11	yeah it (+), and i (−), play (+), no no (+), depression (−)
**Support Vector Machine**	0.75 (0.69–0.81)	9	and (−), yeah it (+), play, no no (+), depression (−)
**Training Data: Non-Mentally Ill Controls, Controls with Mental Illness and Suicidal**			
**Extreme Gradient Boosting**	0.72 (0.65–0.79)	22	and i, anymore, because of, college, depression
**Logistic Regression**	0.72 (0.66–0.79)	27	at my (−), you (+), yeah it (+), attempt (−), college and (−)
**Support Vector Machine**	0.72 (0.65–0.78)	27	you (+), yeah it (+), at my (−), attempt (−), college and (−)

Note: Feature importance was determined from the training data and their root has replaced words (e.g., “am” is the first-person singular version of the verb “be”). Logistic regression and the support vector machine’s feature weights were positive or negative, indicating whether these features influenced the model’s prediction towards the case (+) or control (−). Extreme gradient boosting models’ feature importance is always positive and reflects how frequently a feature was used to make decisions.

**Table 6:** Summary of therapist suicidal risk scores, participants, and sessions.

Therapist	No. of Participants	No. of Sessions	No. of Cases	Average Suicidal Risk Score (SD)	Suicidal Risk Score Range (Min–Max)
A	15	66	2	14.4 (3.1)	8–26
B	14	54	9	6.9 (8.7)	1–51
C	9	67	36	11.2 (7.3)	4–43
D	6	26	10	12.2 (13.5)	3–70
E	5	16	3	10.9 (13.2)	3–54
F	4	18	3	10.2 (3.4)	6–16
G	3	9	1	4.3 (1.9)	2–8
H	2	4	1	9.8 (3.0)	7–14
I	1	5	5	16.4 (7.1)	7–24
J	1	2	0	13 (7.1)	8–18
**All**	**60**	**267**	**70**	**11.0 (8.3)**	**1–70**

Note: Cases are defined as item 9 scores > 0 or answering “yes” to item 12 on the PHQ-A.

## Supplementary Materials

All data that does not contain protected health information that may identify individual patients is found within the paper and its supporting information file(s), available online at https://www.mdpi.com/1660-4601/17/21/8187/s1. Data submitted with this manuscript includes Table S1: questionnaire responses to the PHQ-A and C-SSRS, and the clinicians’ recorded actions and impression ratings of their clients’ suicidal state used in analysis. The speech samples and transcripts used in the analysis contain personal information that can be used to identify individuals. Requests for data can be made to research@clarigenthealth.com.

## Abbreviations

ACT	Adolescent Controlled Trial
AUC	area under receiver operating characteristic curve
CCHMC	Cincinnati Children’s Hospital Medical Center
CI	confidence intervals
CSSRS	Columbia Suicide Severity Rating Scale
ED	emergency department
LR	logistic regression
MHP	mental health professional
ML	machine learning
NLP	natural language processing
PCH	Princeton Community Hospital
PHQ-A	Patient Health Questionnaire modified for Adolescents
STM	Suicidal Though Markers Study
SVD	singular value decomposition
SVM	support vector machine
UCMC	University of Cincinnati Medical Center
UQ	Ubiquitous Questionnaire
XAI	explainable artificial intelligence
XGB	extreme gradient boosting
